# Aparatos ortodónticos fijos y el sistema de alineadores transparentes: una revisión comparativa

**DOI:** 10.21142/2523-2754-1202-2024-198

**Published:** 2024-06-27

**Authors:** Harvy Yassbeck Cárdenas Machuca, Henry Josue Granda Reyes, Xiomara Nicole Marchena Gómez, Luisa Andrea Sierra Carbajal, Luciano Carlos Soldevilla Galarza, Manuel Antonio Mattos-Vela

**Affiliations:** 1 Universidad Nacional Mayor de San Marcos, Facultad de Odontología. Lima, Perú. harvy.cardenas@unmsm.edu.pe , henry.granda@unmsm.edu.pe , xiomara.marchena@unmsm.edu.pe , luisa.sierra@unmsm.edu.pe , lsoldevillag@unmsm.edu.pe , mmattosv@unmsm.edu.pe Universidad Nacional Mayor de San Marcos Universidad Nacional Mayor de San Marcos Facultad de Odontología Lima Peru harvy.cardenas@unmsm.edu.pe henry.granda@unmsm.edu.pe xiomara.marchena@unmsm.edu.pe luisa.sierra@unmsm.edu.pe lsoldevillag@unmsm.edu.pe mmattosv@unmsm.edu.pe

**Keywords:** soportes ortodóncicos, aparatos ortodóncicos removibles, ortodoncia, Orthodontic brackets, removable orthodontic appliances, orthodontics

## Abstract

Una de las mayores controversias de la ortodoncia actual es determinar el aparato a utilizar, ya que hoy en día los pacientes buscan mejores resultados en tiempos más cortos, además de anteponer la estética. Objetivo: Comparar los beneficios y las desventajas que se presentan al utilizar aparatos ortodónticos fijos y alineadores transparentes. Materiales y métodos: Se realizó una investigación y recopilación de información bibliográfica especializada en el tema, en buscadores científicos como PubMed, SciELO y Web of Science, entre los años 1991 y 2023. Los trabajos de investigación estuvieron relacionados con los efectos del uso de aparatos ortodónticos fijos en comparación con alineadores transparentes. Resultados: La revisión se realizó en 53 artículos encontrados que cumplieron los criterios de selección. Conclusión: Los aparatos ortodónticos fijos son mejores en casos complejos, son más precisos y tienen menos probabilidades de recidiva; los alineadores transparentes son más estéticos, la higiene es más efectiva y disminuye la densidad ósea del cóndilo mandibular.

## Abstract (en)

One of the biggest controversies in current orthodontics is determining the appliance to use, since today patients seek better results in shorter times, in addition to putting aesthetics first. Objective: compare the benefits and disadvantages that arise when using fixed orthodontic appliances and transparent aligners. Materials and Methods: An investigation and compilation of specialized bibliographic information on the topic was carried out in scientific search engines such as PubMed, SciElo and Web of Science between the years 1991 to 2023, focused on research work related to the effects of the use of orthodontic appliances. fixed compared to clear aligners. Results: The review was carried out based on 53 articles found that met the selection criteria. Conclusion: Fixed orthodontic appliances are better in complex cases, they are more precise and less likely to relapse; Transparent aligners are more aesthetic, hygiene is more affective and the bone density of the mandibular condyle decreases.

## INTRODUCCIÓN

Los aparatos ortodónticos son dispositivos utilizados en odontología para corregir problemas esqueléticos maxilares, así como de alineación y posición de los dientes y la mandíbula. Estos dispositivos desempeñan un papel crucial en la ortodoncia, una rama de la odontología dedicada a mejorar la estética y la función del sistema estomatognático. Dos categorías principales de los aparatos ortodónticos son los aparatos ortodónticos fijos y los removibles, cada uno con características y aplicaciones específicas. Los aparatos ortodónticos fijos son ideales para tratar problemas más complejos de alineación dental, ya que pueden ejercer fuerzas constantes y controladas para mover los dientes a lo largo del tiempo. Los brackets se fijan en cada diente, mientras que los alambres se conectan a través de ellos, lo que permite ajustes regulares por parte del especialista ^1^^,^^2^.

Por otro lado, los aparatos ortodónticos removibles son dispositivos que el paciente puede retirar y volver a colocar, como placas activas, retenedores o alineadores transparentes. Estos aparatos son adecuados para casos menos complejos de alineación dental y, a menudo, se utilizan en pacientes en crecimiento, ya que se pueden ajustar con el tiempo, a medida que los dientes cambian de posición. Los retenedores, por ejemplo, se usan para mantener los resultados de un tratamiento previo, mientras que los alineadores transparentes son una opción para adultos que desean corregir problemas menores de alineación de manera discreta ^2^^,^^3^.

Actualmente, a pesar de los diversos estudios en la ortodoncia, en la literatura todavía existen algunos desafíos e incógnitas sobre el mecanismo de acción, el tiempo de duración y la efectividad de los aparatos ya mencionados. Asimismo, se desconoce el impacto sobre la salud (alteraciones de la musculatura facial o ATM, problemas periodontales, control de la dimensión vertical) y su posible implementación con las tecnologías de impresión dental, como el CAD CAM o escáner intraoral ^4^^-^^7^. 

El propósito del estudio fue comparar los beneficios y desventajas, con relación a la efectividad, el mantenimiento y las repercusiones en tejidos blandos y duros que se presentan al utilizar aparatos ortodónticos fijos y alineadores transparentes.

## MATERIALES Y MÉTODOS

**Figura 1:**
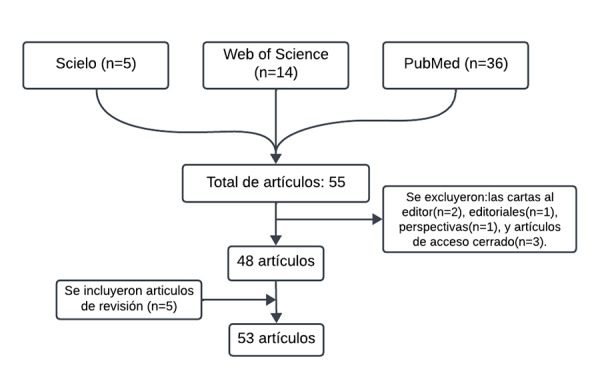
Diagrama de flujo de la selección de artículos

Se realizó una revisión bibliográfica desde 1991 hasta 2023 en las bases de datos: SciELO, PubMed y Web of Science. Al realizar la búsqueda, se emplearon términos como “alineadores transparentes”, “aparatos ortodónticos fijos” y “ortodoncia”. El total de artículos encontrados fue de 48 en español e inglés. Se excluyeron las cartas al editor, editoriales, perspectivas y los artículos de acceso cerrado. Por otro lado, se incluyeron 5 artículos que, a pesar de su antigüedad, eran relevantes, por lo que, finalmente, se consideraron 53 estudios (Figura 1). 

## REVISIÓN DE LA LITERATURA

### Aparatos ortodónticos fijos

También denominados brackets, son de mayor elección debido a su disponibilidad y durabilidad. Suelen ser de acero inoxidable, por su alta rigidez, biocompatibilidad y resistencia a la corrosión. Otros materiales utilizados son la cerámica y el plástico ^5^^,^^6^.

Los brackets cerámicos se caracterizan por su alta resistencia y calidad estética en comparación con los de materiales metálicos; sin embargo, tienen ciertas desventajas tales como su alto costo o fragilidad ^5^^,^^7^^,^^8^.

Otro tipo de brackets son los de autoligado que producen una fricción significativamente menor a la de los metálicos, por lo que necesitan de menor fuerza para generar movilidad en los dientes ^5^^,^^9^.

Por otro lado, se encuentran los brackets linguales, que se diferencian por estar adheridos a la parte posterior de los dientes. Son preferidos por la estética; no obstante, pueden generar laceraciones en la lengua, acumulación de placa y mayor tiempo de consulta por dificultades de instalación ^5^^,^^10^.

Ventajas. Las ventajas de los aparatos fijos están más centradas en los resultados del tratamiento. Por ejemplo (Tabla 1), generan mejores movimientos para extruir o intruir los dientes anteriores ^11^; sus efectos son de largo plazo y más precisos ^12^^,^^13^; a la hora de tratar la mordida profunda generan cambios más notorios ^14^; existe una menor recidiva dentro de los primeros 3 años después de terminar el tratamiento ^2^; la biomecánica puede corregirse en cada visita, según la respuesta de las activaciones realizadas en la cita anterior; y la extrusión y rotación de caninos es muy sencilla ^15^. Además, evitan ciertos riesgos. Por ejemplo, en los incisivos superiores, tanto brackets convencionales como autoligados, no presentan reabsorción radicular ^16^, y no generan una disminución ósea en el cóndilo ^17^. Asimismo, son mucho más rentables en comparación con los aparatos ortodónticos innovadores, ya que sus tratamientos presentan mejores resultados en casos más complejos ^18^. 

**Tabla 1:** Ventajas y desventajas de los aparatos fijos y de los alineadores transparentes

	Aparatos ortodónticos fijos	Alineadores transparentes
Ventajas	Existe una menor recidiva entre 1 y 3 años después del tratamiento (2).	Soluciona las mordidas abiertas leves anteriores en adultos (1, 3).
	Menor precio (18).	Mayor precisión en la constricción lingual (44).
	En los incisivos superiores, casi no presentan reabsorción radicular (9).	Menor duración del tratamiento en maloclusiones de leves a moderadas (2, 3, 45).
	Genera mejores movimientos para extruir o intruir los dientes anteriores (11).	Mayor comodidad a la hora de masticar y comer (39).
	Presentan efecto positivo a largo plazo y son más precisos (12).	Menor retención de placa bacteriana y menor reacción inflamatoria de la gingival (19).
	Sin visualización de disminución ósea en el cóndilo (16).	Mayor estética durante el tratamiento, con mejores resultados visuales solo en casos simples (29).
	Cambios más notorios para cambio de mordida profunda (12, 13).	Menor índice de placa y menor índice de sangrado al sondaje. Se pueden retirar para el correcto lavado (21, 23, 40).
	La biomecánica puede corregirse en cada visita, según la respuesta de las activaciones realizadas en la cita anterior. La extrusión y rotación de caninos es muy sencilla (14).	El tratamiento de la mordida abierta dental es más propenso a ser exitoso (15).
	Presenta una ligera alteración de la actividad muscular (52)	Mejor salud periodontal, menor recuento de bacterias orales, reabsorción radicular menos grave, menos molestias y duración más corta (40-42).
	--------------------------	Eficaz para distalizar los molares superiores con un mejor control de la dimensión vertical, la rotación del plano oclusal y la extrusión de los molares (43).
Desventajas	Mayor dolor en los dientes anteriores, en la mandíbula y dentogingival (23-25).	Las rotaciones no son lo suficientemente sustanciales como para disminuir el ángulo MP-MnOP (1).
	Requieren más analgésicos (26-28).	La mordida profunda asociada al espaciamiento es más difícil de tratar (15).
	Malestar en las primeras semanas (15, 28).	Mayor recidiva (11).
	Mayor frecuencia de emergencias relacionadas con la ortodoncia por ingestión accidental de aparatos o aditamentos, alambre protruido, desprendimiento de bandas, arcos desplazados, etc. (30).	Ausencia del asentamiento oclusal adecuado; requiere un alto nivel de disciplina y compromiso del paciente, dificultad del control vertical y de inclinación vestibular-lingual de los incisivos durante la retracción anterior para el cierre de espacios, lo que provoca inclinación lingual de incisivos, aumenta la sobremordida y exposición incisal, y no favorece movimientos como la extrusión y rotación de caninos (15).
	Deficiencias en el control del movimiento de los dientes, difícil motivación del paciente (6, 18).	Disminución de la densidad ósea en el cóndilo mandibular (17).
	Es necesario una mayor cantidad de cepillado. Presenta mayor irritabilidad gingival, menor higiene bucal, mayor acumulación de placa y mayor susceptibilidad a caries (19-21).	Menos precisos con la rotación de caninos y premolares, intrusión de incisivos mandibulares y traslación a espacios de extracción. Dificultad para corregir maloclusiones comunes. Requieren más de un escaneo de refinamiento para verificar la precisión del movimiento dental (46-48).
	Dificultad a la hora de la limpieza, presenta mayor índice de sangrado al sondaje (18).	Los pacientes con maloclusiones simples requieren 4 u 8 meses más de tiempo (41).
	Se protruyen más los labios durante el tratamiento (53).	Un costo más elevado (18).
	Si el paciente tiene gingivitis, con el tratamiento puede empeorar la enfermedad (20).	Peor rendimiento en los casos más graves (2).
	Los índices periodontales no son favorables (22).	La extrusión de los incisivos maxilares es menor que con el uso de *brackets* convencionales (21).

Desventajas. Las desventajas se derivan del hecho que sean fijos, lo que puede generar malestar en las primeras semanas ^16^ y que contengan áreas retentivas para la placa bacteriana. También se presenta mayor dificultad a la hora de la limpieza y los pacientes con este tipo de aparatología muestran un incremento en el sangrado al sondaje ^19^. En este caso, es necesario cepillarse más veces y con una mejor técnica, al ser propensos a una mayor acumulación de biofilm, por lo que se puede presentar irritabilidad gingival, menor higiene bucal y mayor susceptibilidad a caries dental ^20^^-^^22^. Si el paciente tiene gingivitis, su condición puede empeorar con los brackets ^21^; asimismo, los índices periodontales no son favorables ^23^ y el dolor es más intenso en los dientes anteriores, en la mandíbula y el sector dentogingival ^23^^-^^25^. Por lo mismo que generan molestias en las primeras semanas, requieren más analgésicos ^26^^-^^28^. Durante el tratamiento, se protruyen más los labios ^29^, la motivación del paciente es difícil ^11^ y es mayor la frecuencia de emergencias por ingestión accidental de aditamentos, alambres protruidos, desprendimiento de bandas, arcos desplazados, etc. ^30^ (Tabla 1). 

Higiene y limpieza. Los largos periodos de fijación de los brackets favorecen un entorno para la formación de placa y la adhesión bacteriana, sobre todo Streptococcus mutans, que influyen en la desmineralización del esmalte ^31^^,^^32^. Frente a ello, la medida preventiva más importante es el cepillado dental efectivo; existen diferentes tipos de diseños promovidos para pacientes con ortodoncia. Pero la aplicación de flúor o agentes antibacterianos también es utilizada para evitar consecuencias a nivel del esmalte o las encías, aunque estos dependen de la frecuencia de atención profesional, así como de la propia higiene del paciente ^33^.

### Alineadores transparentes

Se utilizaban inicialmente para la nivelación y alineación de los arcos ante apiñamiento leve o diastemas; sin embargo, en la actualidad, son usados para casos de moderada o extrema complejidad, debido a las rápidas mejoras tecnológicas en cuanto a materiales y técnicas de producción ^34^^-^^36^. No obstante, existen autores en desacuerdo ante los fracasos en ciertos movimientos dentales ^23^.

Existen dos tipos de alineadores: aquellos fabricados con material termoplástico, a través de configuración manual, y los confeccionados con tecnología CAD-CAM, como el sistema de alineadores transparentes ^37^^,^^38^.

Ventajas. Los alineadores transparentes, a pesar de no ser aparatos fijos, se pueden emplear eficazmente en el tratamiento, sin extracción de piezas dentales, de mordidas abiertas leves anteriores en adultos ^1^^,^^3^. En este caso, son más de índole estético, para mayor comodidad del paciente durante su uso y de cuidado gingival; generan mejores resultados solo en casos simples ^29^; los pacientes no presentan inhibición al momento de hablar, sonreír o interactuar ^20^; son más cómodos después de los ajustes del primer y segundo mes ^26^; permiten una mejor higiene bucal ^11^ y comodidad a la hora de masticar y comer ^39^. Los pacientes presentan un menor índice de placa y sangrado al sondaje, debido a que se pueden retirar para el correcto lavado y esto genera mejores índices periodontales ^21^^,^^23^^,^^40^. Además, el paciente es capaz de tener una mejor limpieza fisiológica y manual de las piezas dentales ^22^; menor reacción inflamatoria de la gingiva ^19^ y recuento de bacterias orales; reabsorción radicular menos grave; pocas molestias y de duración más corta ^40^^-^^42^. Las ventajas en el área de ortodoncia son las siguientes: eficacia para distalizar los molares superiores, con un mejor control de la dimensión vertical, la rotación del plano oclusal y la extrusión de los molares ^43^; mayor propensión al éxito en el tratamiento de la mordida abierta ^15^; mayor precisión en la constricción lingual ^44^ y menor duración del tratamiento en maloclusiones de leves a moderadas ^2^^,^^3^^,^^45^.

Desventajas. Las desventajas se basan más en los resultados; por ejemplo, las rotaciones no son lo suficientemente sustanciales como para disminuir el ángulo plano mandibular a plano oclusal mandibular (MP-MnOP) ^1^; posee una menor precisión en extrusión de incisivos y la precisión en todos los movimientos dentales en general es del 50% ^44^; presenta peor rendimiento en los casos más graves ^2^; los pacientes con maloclusiones simples requieren 4 u 8 meses más de tiempo ^41^; son menos precisos con la rotación de caninos y premolares, intrusión de incisivos mandibulares y traslación a espacios de extracción; presenta mayor dificultad para corregir maloclusiones comunes y requiere más de un escaneo de refinamiento para verificar la precisión del movimiento dental ^46^^-^^48^; hay disminución de la densidad ósea en el cóndilo mandibular ^17^, una mayor recidiva ^11^ y la mordida profunda asociada al espaciamiento es más difícil de tratar ^15^. Por último, presenta ausencia del control de torque o asentamiento oclusal adecuado; requiere un alto nivel de disciplina y compromiso del paciente, ya que son aparatos removibles que deben usarse continuamente; presenta dificulta en el control vertical y de inclinación vestibular-lingual de los incisivos durante la retracción anterior para el cierre de espacios, lo que provoca inclinación lingual de incisivos, aumenta la sobremordida y exposición incisal, y no favorece movimientos como la extrusión de caninos ^15^ (Tabla 1).

Higiene y limpieza. Durante un tratamiento, es fundamental la limpieza e higiene de la aparatología, ya sea fija o removible, ya que esto ayuda a que se reduzcan los riesgos de sufrir de enfermedades periodontales, lesiones cariosas y otras enfermedades bucales ^49^.

Respecto de los aparatos removibles, usualmente, se les pide a los pacientes que se los retiren solo cuando van comer o beber cualquier líquido que no sea agua; sin embargo, algunas personas no cumplen esta indicación, lo cual hace que sus aparatos ortodónticos removibles se tiñan. Para evitarlo, se puede usar desde lo más simple, como el agua destilada, hasta productos dentro del mercado odontológico: cristales de limpieza Invisalign y el limpiador sónico inalámbrico combinado con una tableta Retainer Brite. Asimismo, el secado debe realizarse al aire libre ^35^. Para la limpieza de los alineadores transparentes, otros usan enjuagatorios bucales, polident, ácido acético al 2,5%, NaClO al 0,6% y H2O2 al 3 %; no obstante, estas sustancias podrían afectar algunas propiedades físicas del material con el que se trabajó el alineador transparente ^50^^,^^51^.

## DISCUSIÓN

Algunos autores mencionan que los aparatos ortodónticos fijos pueden mejorar la mordida abierta, pero, como consecuencia, empeora la dimensión vertical ^1^^,^^4^^,^^5^^,^^7^^,^^43^. En consecuencia, se opta por el uso de alineadores transparentes solo en casos de mediana o simple complejidad; aunque en casos complejos puede tomar mucho más tiempo el tratamiento ^2^. Por otro lado, la mayoría de los autores concuerda en que la nivelación y rotación de caninos y premolares no es eficiente con el uso de los alineadores transparentes; sin embargo, se encontró que, con el uso de accesorios adicionales novedosos, son capaces de mejorar con eficacia lo señalado, según Papadimitriou et al. ^2^^,^^3^.

El tema de la estética es uno de los problemas que más afecta a las personas cuando van a elegir entre un aparato ortodóntico fijo y los alineadores transparentes. Mundhada et al. ^21^ señala que el uso de los aparatos ortodónticos fijos linguales mejora bastante la estética, pero las desventajas están referidas a la inhibición del desarrollo dental, la interrupción dental, la dificultad a la hora de colocar los brackets, heridas en la lengua, mayor presencia de biofilm dental, irritación gingival y mayor tiempo de consulta ^5^^,^^20^^,^^21^.

Otro problema de los aparatos ortodónticos fijos es la higiene, lo que afecta directamente la salud periodontal. En la revisión, se encontró que los alineadores transparentes tienen una gran ventaja cuando se trata de la salud periodontal. Este resultado coincide con el estudio de Abatte et al. ^19^, en el cual se halló que los adolescentes tratados con aparatos removibles muestran un mejor cumplimiento de la higiene bucal, menos placa y menos reacciones inflamatorias gingivales que sus pares con aparatos fijos. Sin embargo, Lu Haili et al. ^21^, a pesar de tener resultados similares, recomiendan que la información necesita ser confirmada por más ensayos controlados aleatorios ^19^^,^^21^^,^^22^.

En lo que respecta al dolor, se encontró que los aparatos ortodónticos fijos generan una mayor disconformidad en los pacientes debido al malestar en los dientes anteriores y la mandíbula. Asimismo, se mencionó que los pacientes que usaron este tipo de aparatología ortodóntica tuvieron que consumir una mayor cantidad de analgésicos. White et al. ^26^, en un ensayo aleatorizado ciego y prospectivo, registraron el nivel de malestar en reposo, al masticar y al morder, así como el consumo de analgésicos y alteraciones del sueño en un total de 41 pacientes con alineadores removibles y fijos; como resultado obtuvieron que, al inicio, ambas modalidades de tratamiento mostraron niveles similares de malestar. Asimismo, los pacientes del grupo de aparatos fijos tradicionales informaron una incomodidad significativamente mayor que los pacientes del grupo de alineadores removibles durante la primera semana de tratamiento activo; por ello, un mayor porcentaje de pacientes en el grupo de aparatos fijos informaron haber tomado analgésicos durante la primera semana para el dolor dental ^26^^,^^28^.

Por otro lado, según Tamer et al. ^37^, los alineadores transparentes indican un menor desarrollo de lesiones de manchas blancas durante el tratamiento en comparación con los aparatos fijos, pero mencionan, en cuanto a la reabsorción radicular, que ambos aparatos lo poseen como riesgo asociado. Sin embargo, según nuestra revisión, se menciona que los alineadores transparentes poseen menor grado de reabsorción radicular ^40^^-^^42^.

## CONCLUSIONES

• Los aparatos ortodónticos fijos son más rentables y funcionan a largo plazo. Además de ser utilizados en casos de mayor complejidad, son más precisos, no disminuyen la densidad ósea del cóndilo mandibular, no se presenta reabsorción radicular y es menos probable la recidiva.
• Los alineadores transparentes son más estéticos, facilitan la higiene, retienen menor cantidad de bacterias, emplean menor tiempo en casos leves y moderados y casos de mordida abierta.
• Los aparatos ortodónticos fijos no son removibles, lo cual genera dificultad al momento de la higiene bucal, áreas que retienen biofilm, mayor susceptibilidad a la caries y enfermedad periodontal, mayor cantidad de accidentes y dolor intenso en las primeras semanas.
• Los alineadores transparentes poseen menor precisión en movimiento dental, en casos graves tiene bajo rendimiento, son menos precisos para rotaciones dentales, disminuyen la densidad ósea del cóndilo mandibular, es más probable la recidiva y requieren compromiso del paciente.

## References

[1] Moshiri S, Araújo EA, McCray JF, Thiesen G, Kim KB (2017). Cephalometric evaluation of adult anterior open bite non-extraction treatment with Invisalign. Dental Press J Orthod.

[2] Papadimitriou A, Mousoulea S, Gkantidis N, Kloukos D (2018). Clinical effectiveness of Invisalign(r) orthodontic treatment a systematic review. Prog Orthod.

[3] Kau CH, Soh J, Christou T, Mangal A (2023). Orthodontic Aligners Current Perspectives for the Modern Orthodontic Office. Medicina.

[4] Meade M, Weir T (2023). Treatment planning protocols with the Invisalign appliance an exploratory survey. Angle Orthod.

[5] Mundhada V, Jadhav V, Reche A (2023). A Review on Orthodontic Brackets and Their Application in Clinical Orthodontics. Cureus.

[6] Buschang P, Chastain D, Keylor C, Crosby D, Julien K (2019). Incidence of white spot lesions among patients treated with clear aligners and traditional braces. Angle Orthod.

[7] Menéndez C, Menéndez M, Aguilar A, Gómez G, Carreño J, Khaldy H (2022). Salivary Markers of Oxidative Stress in Patients Undergoing Orthodontic Treatment with Clear Aligners versus Self-Ligating Brackets A Non-Randomized Clinical Trial. J Clin Med.

[8] Pasha A, Vishwakarma S, Narayan A, Vinay K, Shetty S, Roy P (2015). Comparison of Frictional Forces Generated by a New Ceramic Bracket with the Conventional Brackets using Unconventional and Conventional Ligation System and the Self-ligating Brackets An In Vitro Study. J Int Oral Health.

[9] Morales I, Gandía J, Cobo J, Vela A, Bellot C (2020). Arch expansion with the Invisalign system Efficacy and predictability. PLoS One.

[10] Khanpayeh E, Jafari A, Tabatabaei Z (2014). Comparison of salivary Candida profile in patients with fixed and removable orthodontic appliances therapy. Iran J Microbiol.

[11] Ke Y, Zhu Y, Zhu M (2019). A comparison of treatment effectiveness between clear aligner and fixed appliance therapies. BMC Oral Health.

[12] Kadioglu M, Cakmak B, Altunal E, Rubendiz M (2023). Evaluation of Orthodontic Treatment Method Preferences of Dentistry Students, Dentists and Orthodontic Residents. Turk J Orthod.

[13] Capistrano A, Cordeiro A, Siqueira D, Capelozza L, Cardoso M, Almeida R (2014). From conventional to self-ligating bracket systems is it possible to aggregate the experience with the former to the use of the latter?. Dental Press J Orthod.

[14] Henick D, Dayan W, Dunford R, Warunek S, Al T (2021). Effects of Invisalign (G5) with virtual bite ramps for skeletal deep overbite malocclusion correction in adults. Angle Orthod.

[15] Machado R (2020). Space closure using aligners. Dental Press J Orthod.

[16] Turner S, Harrison J, Sharif FN, Owens D, Millett D (2021). Orthodontic treatment for crowded teeth in children. Cochrane Database Syst Rev.

[17] Ertugrul B, Veli I (2022). Evaluating the effects of orthodontic treatment with clear aligners and conventional brackets on mandibular condyle bone quality using fractal dimension analysis of panoramic radiographs. J Stomatol Oral Maxillofac Surg.

[18] Feu D, Catharino F, Duplat CB, Capelli J (2012). Esthetic perception and economic value of orthodontic appliances by lay Brazilian adults. Dental Press J Orthod.

[19] Abbate G, Caria M, Montanari P, Mannu C, Orrù G, Caprioglio A (2015). Periodontal health in teenagers treated with removable aligners and fixed orthodontic appliances. J Orofac Orthop.

[20] Azaripour A, Weusmann J, Mahmoodi B, Peppas D, Gerhold A, Van C (2015). Braces versus Invisalign(r) gingival parameters and patients' satisfaction during treatment: a cross-sectional study. BMC Oral Health.

[21] Lu H, Tang H, Zhou T, Kang N (2018). Assessment of the periodontal health status in patients undergoing orthodontic treatment with fixed appliances and Invisalign system A meta-analysis. Medicine.

[22] Miethke R, Vogt S (2005). A comparison of the periodontal health of patients during treatment with the Invisalign system and with fixed orthodontic appliances. J Orofac Orthop.

[23] Mulla Issa FK, Mulla Issa ZK, Rabah A, Hu L (2020). Periodontal parameters in adult patients with clear aligners orthodontics treatment versus three other types of brackets A cross-sectional study. J Orthodont Sci.

[24] Antonio L, Montero J, Garcovich D, Alvarado M, Albaladejo A, Alvarado A (2021). Comparative Analysis of Periodontal Pain According to the Type of Precision Orthodontic Appliances Vestibular, Lingual and Aligners. A Prospective Clinical Study. Biology (Basel).

[25] Noll D, Mahon B, Shroff B, Carrico C, Lindauer S (2017). Twitter analysis of the orthodontic patient experience with braces vs Invisalign. Angle Orthod.

[26] White DW, Julien KC, Jacob H, Campbell PM, Buschang PH (2017). Discomfort associated with Invisalign and traditional brackets A randomized, prospective trial. Angle Orthod.

[27] Almasoud N (2018). Pain perception among patients treated with passive self-ligating fixed appliances and Invisalign(r) aligners during the first week of orthodontic treatment. Korean J Orthod.

[28] Antonio L, Montero J, Albaladejo A, Oteo M, Alvarado A (2020). Pain and Oral-Health-Related Quality of Life in Orthodontic Patients During Initial Therapy with Conventional, Low-Friction, and Lingual Brackets and Aligners (Invisalign) A Prospective Clinical Study. J Clin Med.

[29] Liao T, Fang J, Wang I, Huang C, Chen H, Yen T (2022). Characteristics and Dental Indices of Orthodontic Patients Using Aligners or Brackets. Int J Environ Res Public Health.

[30] Gou Y, Ungvijanpunya N, Chen L, Zeng Y, Ye H, Cao L (2022). Clear aligner vs fixed self-ligating appliances Orthodontic emergency during the 2020 coronavirus disease 2019 pandemic. Am J Orthod Dentofacial Orthop.

[31] Rosenbloom RG, Tinanoff N (1991). Salivary Streptococcus mutans levels in patients before, during, and after orthodontic treatment. Am J Orthod Dentofacial Orthop.

[32] Willmot D (2008). White Spot Lesions After Orthodontic Treatment. Semin Orthod.

[33] Schatzle M, Imfeld T, Sener B, Schmidlin P (2009). In vitro tooth cleaning efficacy of manual toothbrushes around brackets. Eur J Orthod.

[34] Putrino A, Barbato E, Galluccio G (2021). Clear Aligners Between Evolution and Efficiency-A Scoping Review. Int J Environ Res Public Health.

[35] Kuncio D, Maganzini A, Shelton C, Freeman K (2007). Invisalign and traditional orthodontic treatment postretention outcomes compared using the American Board of Orthodontics objective grading system. Angle Orthod.

[36] Guntaka P, Kiang K, Caprio R, Parry G, Padwa B, Resnick C (2023). Do patients treated with Invisalign have less swelling after orthognathic surgery than those with fixed orthodontic appliances. Am J Orthod Dentofacial Orthop.

[37] Tamer I, Oztas E, Marsan G (2019). Orthodontic Treatment with Clear Aligners and The Scientific Reality Behind Their Marketing A Literature Review. Turk J Orthod.

[38] Lagravère M, Flores C (2005). The treatment effects of Invisalign orthodontic aligners a systematic review. J Am Dent Assoc.

[39] Flores C, Brandelli J, Pacheco C (2018). Patient satisfaction and quality of life status after 2 treatment modalities Invisalign and conventional fixed appliances. Am J Orthod Dentofacial Orthop.

[40] Mummolo S, Tieri M, Nota A, Caruso S, Darvizeh A, Albani F (2020). Salivary concentrations of Streptococcus mutans and Lactobacilli during an orthodontic treatment An observational study comparing fixed and removable orthodontic appliances. Clin Exp Dent Res.

[41] Lin E, Julien K, Kesterke M, Buschang P (2022). Differences in finished case quality between Invisalign and traditional fixed appliances. Angle Orthod.

[42] Li Y, Deng S, Mei L, Li Z, Zhang X, Yang C (2020). Prevalence and severity of apical root resorption during orthodontic treatment with clear aligners and fixed appliances a cone beam computed tomography study. Prog Orthod.

[43] Lione R, Balboni A, Di Fazio V, Pavoni C, Cozza P (2022). Effects of pendulum appliance versus clear aligners in the vertical dimension during Class II malocclusion treatment a randomized prospective clinical trial. BMC Oral Health.

[44] Haouili N, Kravitz N, Vaid N, Ferguson D, Makki L (2020). Has Invisalign improved A prospective follow-up study on the efficacy of tooth movement with Invisalign. Am J Orthod Dentofacial Orthop.

[45] Borda A, Garfinkle J, Covell D, Wang M, Doyle L, Sedgley C (2020). Outcome assessment of orthodontic clear aligner vs fixed appliance treatment in a teenage population with mild malocclusions. Angle Orthod.

[46] Kravitz N, Dalloul B, Zaid Y, Shah C, Vaid N (2023). What percentage of patients switch from Invisalign to braces A retrospective study evaluating the conversion rate, number of refinement scans, and length of treatment. Am J Orthod Dentofacial Orthop.

[47] Karras T, Singh M, Karkazis E, Liu D, Nimeri G, Ahuja B (2021). Efficacy of Invisalign attachments A retrospective study. Am J Orthod Dentofacial Orthop.

[48] Murphy S, Lee S, Scharm J, Kim S, Amin A, Wu TH (2023). Comparison of maxillary anterior tooth movement between Invisalign and fixed appliances. Am J Orthod Dentofacial Orthop.

[49] Olimpio K, Bastos J, Henriques J, Cardoso V, Silva PA, Bardal P (2003). Caries y enfermedad periodontal causadas por tratamiento ortodóntico en ausencia de un programa educativo-preventivo. Rev Odontol Dominic.

[50] Bernard G, Rompré P, Tavares J, Montpetit A (2020). Colorimetric and spectrophotometric measurements of orthodontic thermoplastic aligners exposed to various staining sources and cleaning methods. Head Face Med.

[51] Wible E, Agarwal M, Altun S, Ramir T, Viana G, Evans C (2018). Long-term effects of different cleaning methods on copolyester retainer properties. Angle Orthod.

[52] Dellavia C, Begnoni G, Zerosi C, Guenza G, Khomchyna N, Rosati R (2022). Neuromuscular Stability of Dental Occlusion in Patients Treated with Aligners and Fixed Orthodontic Appliance A Preliminary Electromyographical Longitudinal Case-Control Study. Diagnostics.

[53] White D, Julien K, Jacob H, Campbell P, Buschang P (2017). Discomfort associated with Invisalign and traditional brackets A randomized, prospective trial. Angle Orthod.

